# Exploratory Analysis of Concordance Between Clinician-Collected and Self-Sampled Human Papillomavirus Tests in a Small Cohort of Average- and High-Risk Patients

**DOI:** 10.1089/whr.2024.0004

**Published:** 2024-03-13

**Authors:** Ashley Wong, Rebecca Morgis, Juliette Entenman, Sarah I. Ramirez, Amy L. Hays, Tonya S. Wright, Christina M. Scartozzi, Mack T. Ruffin, Jennifer L. Moss

**Affiliations:** ^1^ Penn State College of Medicine, The Pennsylvania State University, Hershey, Pennsylvania, USA.; ^2^ Department of Family and Community Medicine, Penn State College of Medicine, The Pennsylvania State University, Hershey, Pennsylvania, USA.; ^3^ Department of Family and Community Medicine, Penn State College of Medicine, The Pennsylvania State University, State College, Pennsylvania, USA.; ^4^ Department of Obstetrics and Gynecology, Penn State College of Medicine, The Pennsylvania State University, Hershey, Pennsylvania, USA.; ^5^ Department of Family and Community Medicine, Penn State College of Medicine, The Pennsylvania State University, Reading, Pennsylvania, USA.

**Keywords:** cervical cancer, cancer screening, human papillomavirus (HPV), colposcopy, cancer disparities

## Abstract

**Objectives:**

Cervical cancer screening rates have stagnated, but self-sampling modalities have the potential to increase uptake. This study compares the test characteristics of self-sampled high-risk human papillomavirus (hrHPV) tests with clinician-collected hrHPV tests in average-risk (*i.e.,* undergoing routine screening) and high-risk patients (*i.e.,* receiving follow-up after abnormal screening results).

**Methods:**

In this cross-sectional study, a relatively small cohort of average-risk (*n* = 35) and high-risk (*n* = 12) participants completed both clinician-collected and self-sampled hrHPV testing, along with a brief phone survey. We assessed hrHPV positivity, concordance, positive predictive value (PPV), negative predictive value (NPV), sensitivity, and specificity across both methods (for types 16, 18, or other hrHPV). We also explored the relationship between test concordance and sociodemographic/behavioral factors.

**Results:**

Among average-risk participants, hrHPV positivity was 6% for both test methods (*i.e.,* hrHPV-positive cases: *n* = 2), resulting in reported concordance, PPV, NPV, sensitivity, and specificity of 100%. Among high-risk participants, hrHPV positivity was 100% for clinician-collected tests but only 67% for self-sampled tests, showing varied concordance and sensitivity. Concordance was not associated with sociodemographic or behavioral factors.

**Conclusions:**

Self-sampled hrHPV testing demonstrated high accuracy for average-risk patients in this exploratory study. However, its performance was less consistent in high-risk patients who had already received an abnormal screening result, which could be attributed to spontaneous viral clearance over time. The limited number of participants, particularly HPV-positive cases, suggests caution in interpreting these results. Further research with larger cohorts is necessary to validate these findings and to explore the integration of self-sampled hrHPV testing into routine clinical care, particularly for patients with a history of cervical abnormalities.

**Clinical Trial Registration:**

NCT04591977, NCT04585243.

## Introduction

Cervical cancer burden can be attributed to high-risk human papillomavirus (hrHPV) infection, lack of screening, screening errors, and/or absence of follow-up care.^1,2^ The incidence of cervical cancer in the U.S. has decreased over the last 50 years, with annual rates falling from 13.5 per 100,000 women in 1975 to 6.4 per 100,000 women in 2017,^3^ largely due to the implementation of cervical cancer screening.^4^ However, progress in cervical cancer prevention and screening has stalled.^3^

Current recommendations for cervical cancer screening and follow-up focus on cytology and/or hrHPV testing. The U.S. Preventive Services Task Force (USPSTF) cervical cancer screening recommendations for asymptomatic patients with a cervix (ages 30–65) include (1) with cytology alone every 3 years, (2) with hrHPV testing alone every 5 years, or (3) co-testing with cytology and hrHPV testing every 5 years.^5^ The American Society for Colposcopy and Cervical Pathology recommends colposcopic evaluation of abnormal cervical cancer screening results based on risk profile, allowing for early identification and treatment of precancerous lesions, thereby averting morbidity and mortality from cervical cancer.^5,6^

Despite the benefits of cervical cancer screening, only 80.5% of eligible U.S. women were up to date with cervical cancer screening in 2018.^7^ A Healthy People 2030 objective is to increase screening to 84.3%.^7^ Being out of date with cervical cancer screening may be due to barriers such as feelings of embarrassment, fear of pain, and stigma of cervical cancer associated with sexually transmitted infections.^8,9^ In addition, access to these recommended tests may be limited in rural and underserved communities, especially among communities of color, which have disproportionately higher rates of cervical cancer incidence, morbidity, and mortality.^10^ Furthermore, receiving an abnormal cervical cancer screening result may actually be so aversive, it reduces the likelihood of seeking screenings in the future.^11,12^ Thus, additional strategies are needed to address barriers to cervical cancer screening, particularly among patients with a history of abnormal cervical cancer screenings.

The hrHPV self-sampling is an emerging test modality that has the potential to improve cervical screening rates by overcoming some barriers to screening, including embarrassment and anxiety.^13^ Compared with clinician-collected hrHPV tests, self-sampled hrHPV tests have slightly lower sensitivity and specificity,^14^ but they are noninferior for the detection of CIN2+.^15–17^

The purpose of this study was to assess the concordance and other test characteristics for clinician-collected and self-sampled hrHPV tests among average-risk (*i.e.,* patients attending routine screening) and high-risk (*i.e.,* patients attending colposcopy for follow-up on abnormal screening) patients. Furthermore, we aimed to identify any sociodemographic or behavioral correlates of errors in test performance, which could indicate groups for which self-sampling may be a more or less successful testing modality. These findings have implications for the widespread use of self-sampling as a primary screening method, which could increase screening rates and reduce avoidable morbidity and mortality from cervical cancer.

## Materials and Methods

### Procedures

The full study protocols are available through ClinicalTrials.gov (registrations NCT04591977 for average-risk patients and NCT04585243 for high-risk patients). Recruitment took place between December 2020 and October 2022.

Participants were patients receiving care from Family and Community Medicine or Obstetrics and Gynecology clinics within Penn State Health, a multihospital system serving patients throughout central Pennsylvania. To be eligible, patients had to be between the ages of 30 and 65 years of age, have an intact cervix, and be fluent in English or Spanish. Exclusion criteria included pregnancy, cognitive impairment, incarceration, complete hysterectomy, and history of cervical treatment for abnormal Pap or hrHPV test (*e.g.,* cryotherapy, loop electrosurgical excision procedure). We recruited “average-risk” and “high-risk” patients. Average-risk patients were those scheduled for their routine cervical cancer screening. High-risk patients were those scheduled for colposcopy due to abnormal findings on a cervical cancer screening collected by their provider during a recent usual-care visit.

We used multiple strategies to identify potential participants. We mailed study invitation letters to patients with upcoming “well-woman” appointments, according to manual and automated electronic health record (EHR) chart reviews. In addition, we identified patients through direct clinician referral. The study team reviewed eligibility criteria with interested patients, obtained verbal informed consent, and enrolled participants into the study by phone.

Enrolled participants received, by mail, a copy of the informed consent form as well as the study materials: a hrHPV self-sampling tool, low-literacy instructions, a laboratory processing form, and a preaddressed, postage-paid return mailing envelope. Participants were instructed to collect their sample for the hrHPV test 1 week before or after their scheduled appointment, and then mail the completed tool to the Penn State Health clinical laboratory. The Penn State Health clinical laboratory analyzed the self-sampled hrHPV test using identical procedures as the clinician-collected tests, that is, *via* Roche real-time multiplex polymerase chain reaction (PCR). The test detects DNA that indicates infection with HPV16, HPV18, or other hrHPV types (31, 33, 35, 39, 45, 51, 52, 56, 58, 59, 66, and 68). This self-sampled hrHPV test is a commercial product that is not yet approved by the Food and Drug Administration (FDA) for routine use.

After completing the self-sampled test, each participant completed a brief survey over the phone, and the study team provided them with $15 in compensation for their time. Finally, we accessed participants' EHR charts to extract the results of the clinician-collected tests and select other clinical information, which we entered into the study database.

**FIG. 1.**
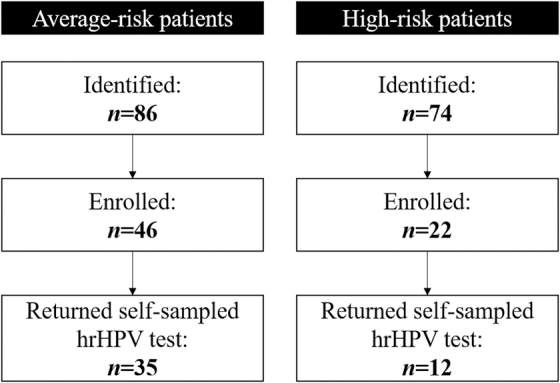
Study flow for participant identification, enrollment, and completion of self-sampled hrHPV test. hrHPV, high-risk human papillomavirus.

Our initial recruitment goals were 197 average-risk participants and 30 high-risk participants, based on stringent assumptions (alpha = 0.01, beta = 0.90, with a paired-sample, noninferiority design, and anticipating discordant results of up to 10%).^18^ However, we terminated enrollment early (after recruiting 46 average-risk and 22 high-risk participants) because of slow accrual and because interim analyses indicated we had achieved adequate statistical power to evaluate our primary outcomes (under assumptions of alpha = 0.05 and beta = 0.80).^18^ For the current study, we limited the analysis to participants who returned a completed hrHPV self-sampling test: 35 average-risk participants (among 46 enrolled, 76%) and 12 high-risk participants (among 22 enrolled, 55%) (Fig. 1).

### Measures

#### Sociodemographic characteristics

From the surveys, we gathered participant characteristics: age group (30–49 years or 50–65 years); race/ethnicity (Hispanic, non-Hispanic White, or other); educational attainment (high school graduate or less, some post-high school training, college graduate or higher, or other); marital status (married or other), and annual household income (<$50,000 or $50,000+).

#### Behavioral factors

From the surveys, we gathered data on factors that had the potential to introduce testing error, including menstruation; use of tampons, spermicide, or vaginal lubricant; vaginal douching; vaginal intercourse; and insertion of anything into the vagina within 48 hours of completing the self-sampled hrHPV test. From the EHR chart review, we also collected measured height and weight, which we used to calculate body mass index (BMI). From study records, we calculated the number of days between collection of samples for the clinician-collected versus self-sampled tests.

#### Screening results

We collected the results of participants' clinician-collected and self-sampled hrHPV tests (separately), which were classified as positive or negative for (1) HPV16, (2) HPV18, or (3) other hrHPV types.

### Statistical analyses

We summarized the sociodemographic characteristics of the sample using descriptive statistics, and we assessed differences between groups using Fisher's exact tests.

The remaining analyses were performed separately for the average- versus high-risk cohorts. We calculated the concordance (yes/no) between clinician-collected and self-sampled hrHPV test results. In addition, we calculated the positive predictive value (PPV), negative predictive value (NPV), sensitivity, and specificity of the self-sampled hrHPV test compared with the clinician-collected hrHPV test (which served as the gold standard).

Finally, we used Wilcoxon rank sum tests and Fisher's exact tests to assess whether concordance between the tests was associated with other study variables (*i.e.,* sociodemographic characteristics or behavioral factors).

Analyses were conducted in SAS version 9.4 and used a two-sided *p*-value of .05. The Penn State College of Medicine Institutional Review Board/Human Subjects Protection Office approved data collection and analysis for this project.

## Results

### Sample characteristics

The study included 35 participants in the average-risk group and 12 in the high-risk group. Participants were predominantly non-Hispanic White (average-risk: 89%; high-risk: 58%), college graduate or higher (average-risk: 77%; high-risk: 50%), and had a household income of $50,000 or more (average-risk: 83%; high-risk: 75%) (Table 1). Average-risk participants were more likely to be older, non-Hispanic White, and married than were high-risk participants (all *p* < 0.05).

**Table 1.** Sociodemographic Characteristics for Average- and High-Risk Participants Who Completed Clinician-Collected and Self-Sampled High-Risk Human Papillomavirus Testing

	Average-risk participants (***n*** = 35)	High-risk participants (***n*** = 12)	***p***
	***n*** (%)	***n*** (%)	
Age group			<0.01
30–49 years	15 (43)	11 (92)	
50–65 years	20 (57)	1 (8)	
Race/ethnicity			0.01
Hispanic	2 (6)	3 (25)	
Non-Hispanic White	31 (89)	7 (58)	
Other	2 (6)	2 (17)	
Educational attainment			0.14
High school graduate	3 (9)	2 (17)	
Some post-high school training	4 (11)	2 (17)	
College graduate or higher	27 (77)	6 (50)	
Other	1 (3)	2 (17)	
Marital status			<0.01
Married	31 (87)	5 (42)	
Other	4 (11)	7 (58)	
Household income			0.47
<$50,000	4 (11)	2 (17)	
$50,000+	29 (83)	9 (75)	

*p-*Values reflect between-group comparisons assessed with Fisher exact tests.

BMI, body mass index.

### hrHPV self-sampling among average-risk participants

Among average-risk participants, 14% had vaginal intercourse, 3% used a vaginal douche, and 3% put something else in their vagina in the 48 hours before completing the self-sampled hrHPV test; none of the participants reported engaging in the other behaviors assessed. On average, participants had a BMI of 29.3 (range: 17.9–44.1). The average time between the clinician-collected and self-sampled tests was 21 days (range: 4–41 days).

For this group, 6% of the samples from the clinician-collected tests were positive for hrHPV (HPV16: 0/35; HPV18: 0/35; other hrHPV: 2/35) (Table 2). Similarly, 6% of the samples from the self-sampled tests were positive for hrHPV (HPV16: 0/35; HPV18: 0/35; other hrHPV: 2/35). Thus, the concordance, PPV, NPV, sensitivity, and specificity between clinician-collected and self-sampled hrHPV test results were 100% for HPV16, HPV18, and other hrHPV types. Because there was no variability in these outcomes, we could not assess associations between concordance and other study variables.

**Table 2.** Test Characteristics for High-Risk Human Papillomavirus Testing Comparing Self-Sampled Tests Against the Gold Standard of Clinician-Collected Tests, for Average- and High-Risk Participants

	Average-risk participants (***n*** = 35)		
	HPV16	HPV18	Other hrHPV
hrHPV positivity			
Clinician-collected tests, *n* (%)	0 (0)	0 (0)	2 (6)
Self-sampled tests, *n* (%)	0 (0)	0 (0)	2 (6)
Concordance, *n* (%)	35 (100)	35 (100)	35 (100)
PPV	100%	100%	100%
NPV	100%	100%	100%
Sensitivity	100%	100%	100%
Specificity	100%	100%	100%

	High-risk participants (***n*** = 12)		
	HPV16	HPV18	Other hrHPV
hrHPV positivity			
Clinician-collected tests, *n* (%)	2 (17)	2 (17)	9 (75)
Self-sampled tests, *n* (%)	2 (17)	2 (17)	5 (42)
Concordance, *n* (%)	12 (100)	12 (100)	9 (67)
PPV	100%	100%	100%
NPV	100%	100%	43%
Sensitivity	100%	100%	69%
Specificity	100%	100%	100%

hrHPV, high-risk human papillomavirus; NPV, negative predictive value; PPV, positive predictive value.

### hrHPV self-sampling among high-risk participants

Among high-risk participants, 18% were menstruating at the time of the self-sampled test, and 18% had vaginal intercourse in the 48 hours before completing the self-sampled test; none of the participants reported engaging in the other behaviors assessed. On average, participants had a BMI of 29.9 (range: 21.0–47.3). The average time between the clinician-collected and self-sampled test was 71 days (range: 11–212 days).

For this group, by design, 100% of samples from the clinician-collected tests were positive for hrHPV (HPV16: 2/12; HPV18: 2/12; other hrHPV: 9/12 (one sample was positive for both HPV16 and an “other” hrHPV type)) (Table 2). In contrast, 67% of the samples from the self-sampled tests were positive for hrHPV (HPV16: 2/12; HPV18: 2/12; other hrHPV: 5/12; note that one sample was positive for both HPV16 and an “other” hrHPV type). Thus, the overall concordance between clinician-collected and self-sampled hrHPV test results was 67%, with eight participants receiving the same results from the two tests, and four participants receiving different results from the two tests. For HPV16 and HPV18, the concordance, PPV, NPV, sensitivity, and specificity were 100%. For other hrHPV types, the concordance was 67%, PPV was 100%, NPV was 43%, sensitivity was 69%, and specificity was 100%.

Concordance on hrHPV tests among participants in the high-risk group was not associated with any of the sociodemographic characteristics or behavioral factors assessed (all *p* > 0.10). The average time between tests was 66 days (range: 11–212) for participants with concordant results and 81 days (range: 56–91) for participants with discordant results; however, this difference was not statistically significant (*p* = 0.14).

## Discussion

In our evaluation of clinician-collected and self-sampled hrHPV tests, we demonstrated complete concordance (100%) between the two modalities for average-risk participants (*i.e.,* patients attending routine screening) and moderate concordance (67%) for high-risk participants (*i.e.,* patients attending colposcopy after an abnormal cervical cancer screening result). Among the high-risk participants, the discordance between clinician-collected and self-sampled test results was limited to other hrHPV types besides HPV16 and HPV18. We evaluated potential sociodemographic and behavioral factors that could lead to these discrepancies in results, but none of them was associated with concordance. However, for both groups, comparing self-sampled hrHPV tests against the clinician-collected “gold standard,” the PPV and specificity values were 100%. Overall, our findings demonstrate qualified support for self-sampling as a hrHPV testing modality for cervical cancer screening.

Our findings comparing results of self-sampled versus clinician-collected hrHPV tests among average-risk patients attending routine cervical cancer screening resonate with extant literature.^14–17,19^ For example, one meta-analysis combining results from 56 studies demonstrated that analysis of self-sampled hrHPV samples using PCR (the method our laboratory used) is accurate when compared with clinician-collected samples.^19^ It should be noted that, even though the observed test characteristics were all 100% in the average-risk sample, every screening test (including self-sampled and clinician-collected hrHPV tests) is imperfect, and measures of PPV, NPV, sensitivity, and specificity are necessarily “pliable,” that is, dependent on a number of contextual factors, for example, laboratory procedures.^20^

Given these caveats, the clinical implications of self-sampled hrHPV testing could be profound. This modality can provide an alternative to the typical cervical cancer screening involving an invasive procedure in a medical facility, which could increase accessibility, especially among patients who face barriers to care, such as embarrassment^13^ and time constraints,^17^ as well as patients from marginalized populations.^21^

In addition, acceptability of self-sampling for hrHPV testing among patients is very high,^22^ and this testing modality is amenable to large-scale health promotion efforts to augment standard-of-care clinical practice.^23,24^ Thus, self-sampled hrHPV testing holds tremendous promise for increasing cervical cancer screening, and our findings contribute to the growing evidence base demonstrating the accuracy and feasibility of this testing modality.

Notably, however, self-sampled hrHPV test characteristics were suboptimal for high-risk participants who were receiving colposcopy after an abnormal cervical cancer screening. Four of the 12 high-risk participants had discordant results between the clinician-collected versus self-sampled hrHPV tests. This discordance was limited to participants who first tested positive for “other” hrHPV and later tested negative for hrHPV, a pattern that may be explained by clearance of the virus during the time between the two sample collections.^25^ Most hrHPV infections are transient and clear spontaneously within 12–24 months of first detection.^25,26^ Furthermore, clearance may be even faster for “other” hrHPV types compared with hrHPV types 16 and 18.^27^ For high-risk participants in our study with discordant results, an average of 81 days elapsed between sample collections (compared with 66 days for high-risk participants with concordant results), in which time the infection with “other” hrHPV may have cleared spontaneously.

Other factors that can influence clearance of the hrHPV include host genetics, host immune response characteristics, long-term oral contraceptive use, smoking, alcohol consumption, parity, and presence of other sexually transmitted diseases.^26^ Understanding the accuracy of self-sampled hrHPV testing among patients with a history of cervical abnormalities is crucial given existing evidence that this population may be hesitant to engage in guideline-concordant repeat testing^11,12^; additional research is warranted.

In general, the average-risk and high-risk participants were similar to each other, although there were some notable exceptions. Compared with the average-risk participants, the high-risk participants (*i.e.,* those who had already received abnormal results on a routine cervical cancer screening) were younger, less likely to be non-Hispanic White, and less likely to be married. These indicators resonate with other studies demonstrating that the rates of hrHPV infection and cervical cancer are higher among more vulnerable patients.^28,29^

Although studies often focus on access to care as an explanation for these health disparities,^30,31^ this factor likely had a minimal impact on outcomes in our study, because participants (1) were already integrated into a health care system and (2) were provided the self-sampled hrHPV test at no charge. However, participants in the high-risk group were less likely than those in the average-risk group to return the self-sampled hrHPV test (55% vs. 76%, respectively). Understanding why high-risk participants did not complete the self-sampled hrHPV test is critical for developing interventions to improve early detection, follow-up, and treatment to mitigate the burden and disparities of cervical cancer.

### Strengths and limitations of the study

Strengths of our study include inclusion of average-risk and high-risk patients, which allowed us to parse differences in concordance across groups. Previous research has focused on general patient populations, without explicit comparisons between average- and high-risk patients, which is an innovation of the present study. Additionally, concordance and other test characteristics were evaluated across multiple clinically relevant hrHPV outcomes. Limitations include relatively small sample size due to slow participant accrual, although we had adequate power to undertake our main analyses; however, future studies should expand recruitment and enrollment efforts to repeat these analyses on larger, more diverse samples. Because our study design required participants to attend an appointment to obtain the clinician-collected sample, it excluded patients who did not engage in care. These latter patients may be most likely to benefit from the option of self-sampling for hrHPV testing to get up to date with their cervical cancer screening.

## Conclusions

We demonstrated complete concordance (100%) between clinician-collected and self-sampled hrHPV testing for average-risk participants (*i.e.,* patients attending routine cervical cancer screening) and moderate concordance (67%) for high-risk participants (*i.e.,* patients attending colposcopy after an abnormal cervical cancer screening). Future studies should examine whether discordance across tests can be attributed to spontaneous viral clearance. Self-sampling for hrHPV has the potential to bring cervical cancer screening to more women by removing barriers of pelvic exam, scheduling an appointment, and cost, but additional research is needed on ways to optimize accuracy and feasibility of this testing modality across patient populations.

## Abbreviations Used

BMI: body mass index
EHR: electronic health record
FDA: Food and Drug Administration
hrHPV: high-risk human papillomavirus
NPV: negative predictive value
PCR: polymerase chain reaction
PPV: positive predictive value
